# Correlation between interleukins in aqueous humor and vitreous humor of vitreoretinal lymphoma patients

**DOI:** 10.1186/s40662-025-00438-0

**Published:** 2025-06-05

**Authors:** Yurun Liu, Xinyi Zhou, Kaiyu Zhang, Shixue Liu, Ruiwen Li, Yifan Gong, Zhujian Wang, Tingting Jiang, Ting Zhang, Gezhi Xu, Junxiang Gu, Qing Chang

**Affiliations:** 1 Eye Institute and Department of Ophthalmology, Eye & ENT Hospital, Fudan University, 83 Fenyang Rd, Xuhui District, Shanghai, China; 2 Shanghai Key Laboratory of Visual Impairment and Restoration, Shanghai, China; 3 NHC Key Laboratory of Myopia and Related Eye Diseases; Key Laboratory of Myopia and Related Eye Diseases, Chinese Academy of Medical Sciences, Shanghai, China; 4 Department of Nursing, Eye & ENT Hospital, Fudan University, Shanghai, China; 5 Clinical Laboratory, Eye & ENT Hospital, Fudan University, Shanghai, China

**Keywords:** Intraocular lymphoma, Vitreoretinal lymphoma, Cytokine, Interleukin 10, Interleukin 6, Diagnosis

## Abstract

**Background:**

Interleukin detection is helpful in screening vitreoretinal lymphoma (VRL). However, the levels of interleukin in aqueous humor (AqH) can be abnormally low in some cases, leading to underdiagnosis of VRL merely dependent on AqH. The purpose of this study was to investigate the correlation of interleukins between paired AqH and vitreous humor (VH) samples in VRL cases, and to explore potential factors affecting interleukin levels and diagnostic parameters.

**Methods:**

This was a case series study. Reviewed were consecutive biopsy-proven B-cell VRL cases of which adequate paired AqH and VH samples were obtained for the measurement of interleukin 10 (IL-10) and interleukin 6 (IL-6). The correlations of IL-10 and IL-6 between AqH and VH were analyzed. Influences of clinical manifestations on IL levels and positive rates of IL-related parameters in AqH and VH were evaluated, which included AqH IL-10 > 30 pg/mL, VH IL-10 > 65 pg/mL, IL-10/IL-6 ratio > 1, and Interleukin Score for Intraocular Lymphoma Diagnosis (ISOLD) > 0 in both the AqH and VH.

**Results:**

Seventy-four eyes of 64 patients with VRL were included. IL-10 in VH was significantly higher than in AqH (median: 1159.77 vs. 225.74 pg/mL, *P* < 0.001). For both IL-10 and IL-6, the AqH concentrations were positively correlated with VH concentrations in the form of power functions (*P* < 0.001 and *P* < 0.001, respectively). The positive rate of AqH IL-10/IL-6 > 1 (77%) was lower than that of VH IL-10 > 65 pg/mL (91%), VH IL-10/IL-6 > 1 (89%) and VH ISOLD > 0 (91%). Eyes without intraretinal infiltration tended to have lower IL-10 levels in the AqH and VH (median: 141.08 pg/mL vs. 449.10 pg/mL, 825.48 pg/mL vs. 2285.77 pg/mL; *P* = 0.001 and *P* < 0.001, respectively), and lower positive rates of AqH IL-10 > 30 pg/mL (78% vs. 97%, *P* = 0.018) and AqH ISOLD > 0 (76% vs. 97%, *P* = 0.033).

**Conclusions:**

IL-10/IL-6 in AqH may not be as sensitive as the parameters (including IL-10, IL-10/IL-6 and ISOLD) in VH for VRL screening. Cases without intraretinal involvement were less likely to be positive for IL-10 > 30 pg/mL and ISOLD > 0 in AqH; the possibility of VRL should be ruled out more cautiously in these cases.

## Background

Vitreoretinal lymphoma (VRL) is a rare ocular malignancy and is classified as a subtype of the lymphomas of immune-privileged sites [1], of which the majority pathologically belongs to large B-cell lymphomas [2]. VRL is relatively difficult to diagnose because the manifestations can extensively mimic uveitis [3–5]. Therefore, auxiliary examinations are of certain importance in VRL screening.

Due to the B-cell origin in the majority of VRL cases, interleukin 10 (IL-10) and interleukin 6 (IL-6) detection in aqueous humor (AqH) is a less invasive and cost-effective approach to facilitate the differential diagnosis and management of VRL [6, 7]. In AqH samples, IL-10 > 30 pg/mL, ratio of IL-10/IL-6 > 1 [8] and the Interleukin Score for Intraocular Lymphoma Diagnosis (ISOLD) score over certain cutoff value [9, 10] can yield sound sensitivity and specificity.

However, we noticed that IL-10 levels in the AqH may be abnormally lower than that in the vitreous humor (VH) of some VRL cases in practice, which can lead to false negative outcomes in VRL screening. Therefore, figuring out potential factors affecting cytokine levels can help to avoid underdiagnosis of VRL in such circumstances.

In this study, we investigated the correlation of IL-10 and IL-6 levels in paired AqH and VH of VRL patients. We further analyzed the influence of various factors on IL levels and IL-related diagnostic parameters.

## Methods

### Study design

This single-centered case series study was approved by the ethics committee of Fudan University Eye & ENT Hospital (Approval No. 2020119) and conducted in accordance with the tenets of the Declaration of Helsinki. Consecutive cases were reviewed and included if the diagnosis of large B-cell VRL was confirmed via biopsy and undiluted paired AqH and VH samples were acquired for cytokine detection. Exclusion criteria included history of vitrectomy or intravitreal or systemic chemotherapy. The medical history, cytokine data, ultra-wide scanning laser fundoscopy and optical coherence tomography (OCT) images were reviewed.

### Diagnostic vitrectomy and fluid collection

Glucocorticoid, if administrated systemically, was withdrawn at least 2 weeks ahead of vitrectomy. In case of significant lens opacity, phacoemulsification and intraocular lens implantation were performed after the collection of undiluted fluids to avoid possible dilution. Diagnostic vitrectomy was performed through the standard 25-gauge, 3-port pars plana vitrectomy system (Constellation®, Alcon, Fort Worth, TX, USA). First, 100 μL of AqH was obtained through anterior chamber paracentesis; approximate 1.0 mL of undiluted VH was subsequently aspirated via vitrectomy cutter without infusion. Then, the infusion was turned on, and 10 mL of diluted VH was collected. Paired AqH and VH samples of 50 μL were spared for the measurement of IL-10 and IL-6; the rest of the intraocular fluids and cassette fluid were sent for a cytopathological workup immediately.

The cytopathological workup included microscopy of smears and cell pellet sections, as well as molecular examinations consisting of immunoglobulin (Ig) gene rearrangement, immunohistochemistry (IHC) and high-sensitivity cell-free DNA sequencing. Microscope analyses were performed by an experienced cytopathologist at the Fudan University Shanghai Cancer Center. Cytopathology was considered positive when there were typical large basophilic pleomorphic cells with a high nucleus-to-cytoplasm ratio, or atypical dysmorphic cells featuring median-to-large nuclei and nucleoli with at least one positive outcome among the molecular examinations—CD20 staining and high Ki-67 proliferation index in IHC, Ig gene rearrangement, or hits for VRL-related mutations or variations [11].

IL-10 and IL-6 were measured at the clinical laboratory of Fudan University Eye & ENT Hospital with a clinical cytometer (BD FACSCanto II, Becton Dickinson and Company, Franklin Lakes, New Jersey, USA) using a cytometric bead assay kit (12-in-1 human inflammatory cytokines kit, RAISECARE Biotechnology Ltd, Qingdao, Shandong, PRC) approved by the Medical Products Administration for clinical use. Calibration was performed before each batch of assays according to the manual.

### Evaluation of clinical manifestations

**Fig. 1:**
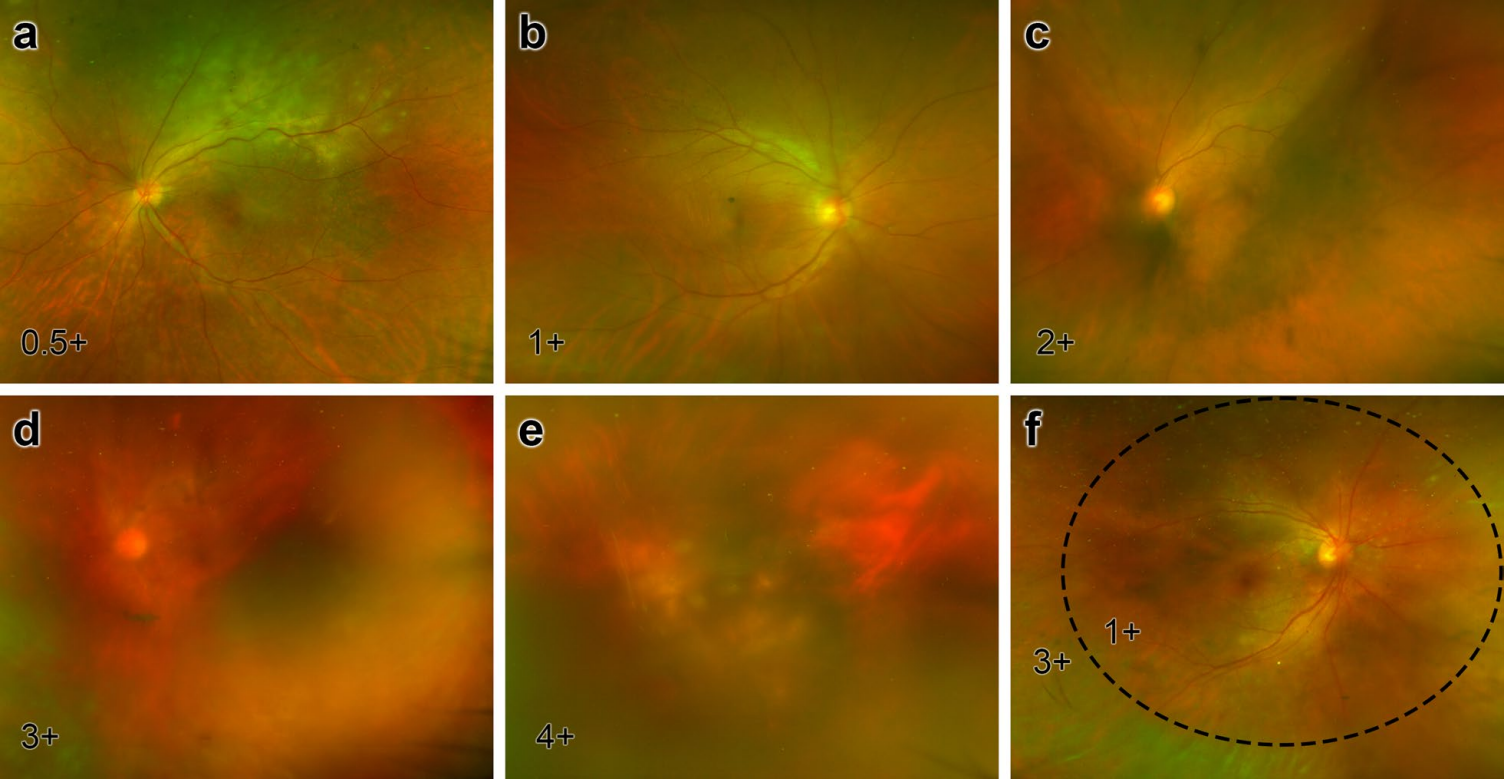
Grading of vitreous haze. **a-e** The vitreous haze was graded from 0.5+ to 4+ as recommended by the Standardization of Uveitis Nomenclature Working Group. **f** For peripherally distributed haze, the intensities for the posterior and peripheral vitreous were graded separately and then averaged. In this eye, the vitreous haze was 1+ within the dashed line but 3+ outside this area; the overall grade then averaged out to 2+. In the case of an average of two contiguous grades (e.g., 2+ and 3+), the overall grade was rounded up or down according to the proportion of the vitreous of the two grades

**Fig. 2:**
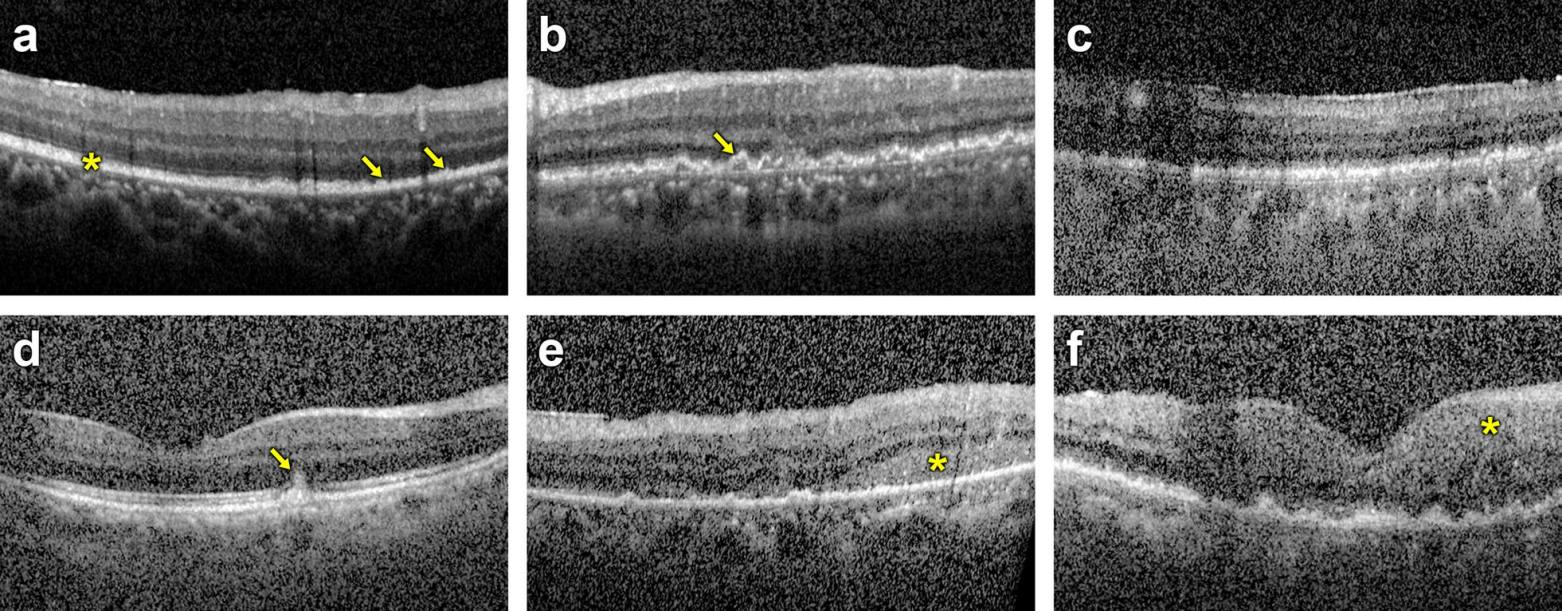
The presence of retinal pigment epithelium (RPE) involvement and intraretinal infiltration was evaluated through optical coherence tomography (OCT). (**a**) RPE thickening (arrow, focal thickening; asterisk, diffuse thickening), (**b**) focal sub-RPE lesions (arrow) and (**c**) diffuse sub-RPE lesions were classified as RPE involvement. (**d**) Focal retinal lesion (arrow), (**e**) diffuse retinal lesion and (**f**) diffuse retinal thickening were classified as intraretinal infiltration. RPE involvement and intraretinal infiltrations could coexist in some eyes (**b**, **d**, **e** and **f**)

The intensity of vitreous haze was graded from 0 to 4 + as recommended by the Standardization of Uveitis Nomenclature Working Group via the latest scanning laser fundoscopy image before vitrectomy; for the eyes in which the haze was restricted to the peripheral vitreous while leaving the posterior vitreous relatively clear, the intensities for the posterior and peripheral vitreous were graded separately and then averaged to better illustrate the overall extent of vitreous haze and cell density (Fig. 1). The presence of retinal involvement was evaluated through OCT (SPECTRALIS HRA + OCT, software version 6.9, Heidelberg Engineering, Heidelberg, Germany); thickening and sub-epithelial lesions of the retinal pigment epithelium (RPE) were classified as RPE involvement (Fig. 2a–c); the presence of intraretinal infiltration was considered if the lesions invaded through the RPE into the neural retina (Fig. 2d–f). In consideration of possible influence of the lens and vitreous on the diffusion of cytokines, recorded were the potential factors including the history of cataract surgery prior to diagnostic vitrectomy, axial length (AL) and age.

### Statistical analysis

Normality of data was evaluated using the Shapiro–Wilk test. Normally distributed continuous variables are presented as mean ± standard deviation with 95% confidence interval (95% CI); non-normally distributed continuous variables are presented as median with range.

The comparison of IL levels between paired AqH and VH samples was conducted using paired Wilcoxon rank-sum test. IL-10 > 30 pg/mL in AqH, IL-10 > 65 pg/mL in VH, and IL-10/IL-6 ratio > 1.0 in the AqH or VH was considered positive [8]. The ISOLD scores for AqH and VH were calculated according to Eqs. (1) and (2), respectively; ISOLD scores over 0 (probability > 50%) were considered positive [9].

1 $${\text{ISOLD}}_{AqH} = - 12.871 + 5.533{\text{ log}}\left( {\left[ {{\text{IL}} - 10} \right] + 1} \right) - 1.614{\text{ log}}\left( {\left[ {{\text{IL}} - 6} \right] + 1} \right)$$

2 $${\text{ISOLD}}_{VH} = - 12.208 + 4.648{\text{ log}}\left( {\left[ {{\text{IL}} - 10} \right] + 1} \right) - 1.669{\text{ log}}\left( {\left[ {{\text{IL}} - 6} \right] + 1} \right)$$

The positive rates of these parameters were compared through Cochran’s Q test and McNemar tests; the outcomes of multiple cross-comparisons were adjusted by the Benjamini–Hochberg method. Least-square-based curve estimation was conducted to evaluate what function the relation of IL-10 or IL-6 between AqH and VH was more likely to be subject to; the R-squared value of each function was computed to reflect the goodness of curve fitting.

To investigate the influence of potential factors on IL levels in AqH, general estimating equation (GEE) was performed; an exchangeable-structured working matrix was used to adjust inner-correlation between the two eyes in bilaterally included patients; the dependent variable was the concentration of each IL in AqH; the independent variables were age, lens status (phakic or IOL), AL and the concentration of the corresponding IL in VH.

To seek potential factors for IL diffusion, we computed the difference in values of IL concentration between the VH and AqH and presented it with a δ prefix (e.g., IL-10 levels in VH minus IL-10 level in AqH is recorded as δ IL-10). We speculated that the diffusion of the cytokines within the eye had reached homeostasis where the concentration would not change over time. According to Fick’s first law of diffusion [see Eq. (3)], the diffusion flux (J) of a certain IL is proportional to the concentration gradient.

3 $${\text{J}} = - {\text{D}} \cdot dC/dx$$

Therefore, the difference values could reflect the difficulty for IL-10 and IL-6 to diffuse from the vitreous cavity to the anterior chamber and thus could help us to figure out potential factors affecting the IL concentration in the AqH. Besides, GEE was conducted to analyze the influence of age, lens status and AL on IL diffusion.

The influence of clinical manifestations on cytokine concentrations in the AqH and VH was analyzed using stepwise linear regression; the dependent variable was the concentration of each cytokine in either the AqH or VH, and the independent variables included the vitreous haze grade, intraretinal infiltration and RPE involvement. For the manifestation of significant effect on IL levels, the eyes were re-grouped according to the presence or absence of this sign; the positive rates of the diagnostic parameters were then compared between the groups to test the effect of a certain sign on the detection rate using Pearson’s Chi-square tests. The positive rates of parameters between eyes with and without the *MYD88*^L265P^ mutation were also compared using the Chi-square tests.

We used SPSS (version 22.0; SPSS Inc., Chicago, IL, USA) for data analysis. *P* values lower than 0.05 indicated statistical significance.

## Results

### Demographics and clinical characteristics

Seventy-four eyes of 64 patients were included, of whom 44 (69%) were female and 20 (31%) were male. The average age was 60 ± 11 (95% CI 57–63) years. The median AL of the eyes was 23.23 (range: 21.54–31.56) mm. Seven (9%) eyes of 7 patients had received phacoemulsification and IOL implantation prior to diagnostic vitrectomy; 67 (91%) eyes of 59 patients were phakic before vitrectomy (Table 1).

**Table 1:** Subject demographics and clinical characteristics

Parameter	Value
Subjects (n = 64)	
Age (mean ± SD, years)	60 ± 11
Gender (female/male)	44/20
Eyes included (n = 74)	
AL (median and range, mm)	23.23, 21.54–31.56
Lens (IOL/phakic)	7/67
*MYD88*^L265P^ (mutant/wild type)	54/20

*SD* = standard deviation; *AL* = axial length; *IOL* = intraocular lens

VH samples from all 74 eyes were positive for cytopathology. Ig gene rearrangement was performed on 20 eyes, and all (20/20, 100%) were positive. High-sensitivity cell-free DNA sequencing was performed for all the 74 eyes, of which *MYD88*^L265P^ mutation was detected in 54 eyes (54/74, 73%; Table 1).

### Comparison between cytokine levels in the AqH and VH

**Fig. 3:**
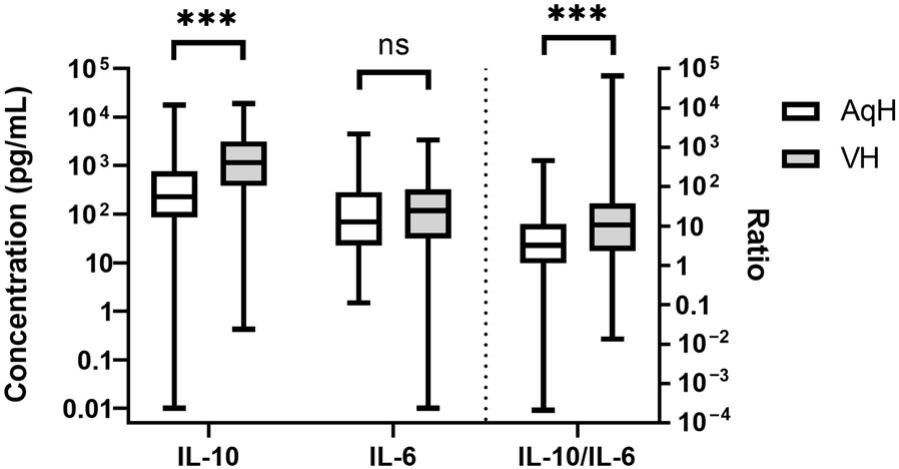
Comparisons of interleukin 10 (IL-10), interleukin 6 (IL-6) and IL-10/IL-6 ratio in aqueous humor (AqH) and vitreous humor (VH). The whisker-box chart shows the levels of IL-10, IL-6 and IL-10/IL-6 ratio in the AqH and VH. The IL-10 level and IL-10/IL-6 ratio in AqH are significantly lower than in VH, while the difference in IL-6 levels between the AqH and VH is of no statistical significance. The boxes indicate the interquartile ranges and the middle lines are the medians; the whiskers show the range of values. ***, *P* < 0.001; ns, not significant

The median IL-10 level was 225.74 (range: 0.01–17,799.72) pg/mL in AqH and 1159.77 (range: 0.42–18,915.42) pg/mL in VH; the median IL-10/IL-6 ratio was 3.27 (range: 0.00–461.99) in AqH and 10.60 (range: 0.01–66,002.00) in VH. IL-10 levels and IL-10/IL-6 ratios in VH were significantly higher than in the AqH (*P* < 0.001 and *P* < 0.001, respectively). No difference in IL-6 level was observed between the AqH and VH (P = 0.191; Fig. 3).

The positive rates of AqH IL-10 > 30 pg/mL, VH IL-10 > 65 pg/mL, AqH IL-10/IL-6 > 1, VH IL-10/IL-6 > 1, AqH ISOLD > 0 and VH ISOLD > 0 were 64/74 (86%), 67/74 (91%), 57/74 (77%), 66/74 (89%), 62/74 (84%) and 67/74 (91%), respectively (Table 2), among which the differences were statistically significant (Cochran’s Q test, *P* = 0.001). Multiple comparisons showed that the positive rate of AqH IL-10/IL-6 > 1 was significantly lower than VH IL-10 > 65 pg/mL, VH IL-10/IL-6 > 1 and VH ISOLD > 0 (McNemar test and Benjamini–Hochberg method; *P* = 0.030, *P* = 0.030, and *P* = 0.030, respectively).

**Table 2:** Positive rate of IL parameters in the AqH and VH

Parameter	^†^Positivity n (%)	Significance of cross comparison^#^				
		AqH IL-10 > 30 pg/mL	AqH IL-10/IL-6 > 1	AqH ISOLD > 0	VH IL-10 > 65 pg/mL	VH IL-10/IL-6 > 1
AqH IL-10 > 30 pg/mL	64 (86%)	–	–	–	–	–
AqH IL-10/IL-6 > 1	57 (77%)	0.345	–	–	–	–
AqH ISOLD > 0	62 (84%)	0.682	0.486	–	–	–
VH IL-10 > 65 pg/mL	67 (91%)	0.680	0.030*	0.450	–	–
VH IL-10/IL-6 > 1	66 (89%)	0.859	0.030*	0.542	> 0.999	–
VH ISOLD > 0	67 (91%)	0.625	0.030*	0.375	> 0.999	> 0.999

*IL* = interleukin; *AqH* = aqueous humor; *VH* = vitreous humor; *IL-10* = interleukin 10; *IL-6* = interleukin 6; *ISOLD* = Interleukin Score for Intraocular Lymphoma Diagnosis

^†^Positivity based on the total number of eyes included in the study (n = 74)

^#^ *P* values of McNemar tests adjusted using the Benjamini–Hochberg method

^*^ *P* < 0.05

**Fig. 4:**
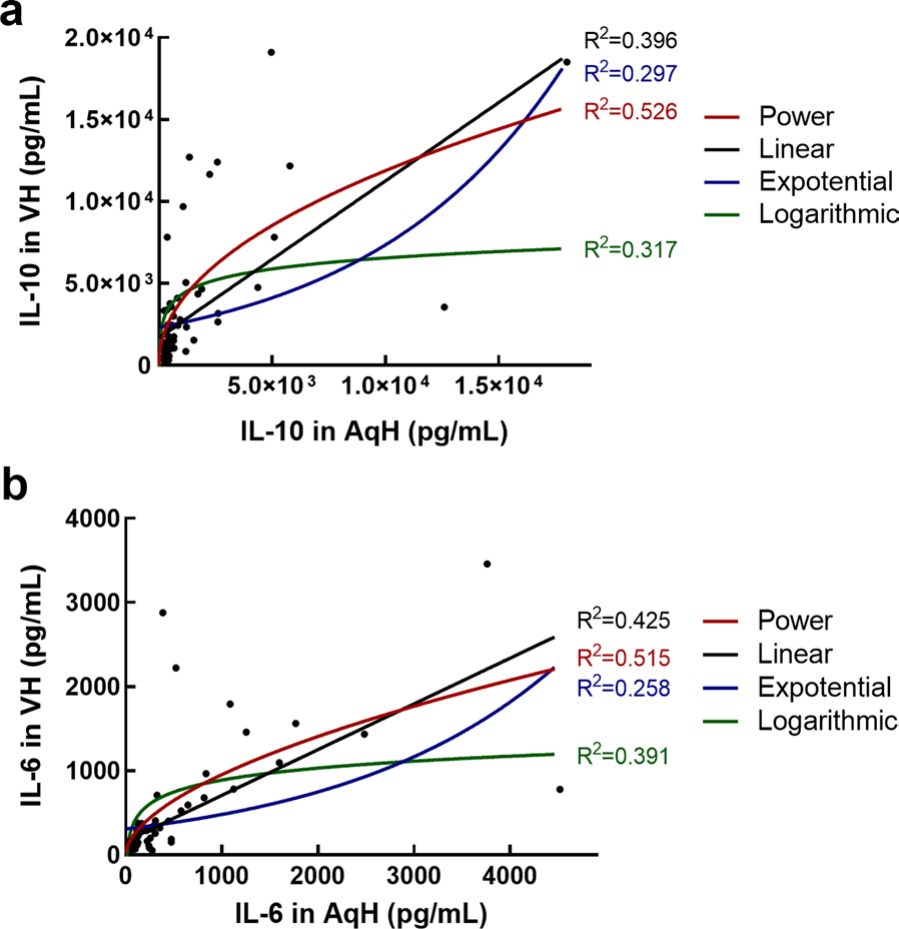
The correlation between the concentrations of interleukin 10 (IL-10) and interleukin 6 (IL-6) in the aqueous humor (AqH) and vitreous humor (VH). The AqH concentrations of both (**a**) IL-10 and (**b**) IL-6 were positively correlated with VH concentrations (*P* < 0.001 and *P* < 0.001, respectively). Curve fitting based on least-square-based regression showed that the goodness of fit (represented by the R-squared value) is the best when a power equation was used for regression. The scatter dots were the data sets of paired IL levels, and the curves were the fitting outcomes of different equations

The correlation between the concentrations of each cytokine in AqH and VH were evaluated via least-square-based regression. For both IL-10 and IL-6, the AqH concentrations were positively correlated with VH concentrations (*P* < 0.001 and *P* < 0.001, respectively); the R-squared values reached the maximum of 0.526 and 0.515, respectively, when a power equation was used for regression (Fig. 4).

### Influences of factors on cytokine levels in the AqH

To investigate possible factors affecting the levels of IL-10 and IL-6 in the AqH, the concentration of each cytokine in the AqH was selected as the dependent variable in GEE; age, lens status, AL and VH concentration were selected as independent variables. The concentrations of both IL-10 and IL-6 in the AqH were most dependent on their concentrations in the VH; age, lens status and AL had little influence on the AqH concentrations of these cytokines (Table 3).

**Table 3:** Correlations of factors on AqH concentration and cytokine diffusion

Dependent variable	Significance of each factor in GEE			
	Age	Lens status	AL	VH concentration
AqH IL-10	0.365	0.973	0.832	0.006*
AqH IL-6	0.992	0.448	0.438	0.003*
δ IL-10	0.796	0.561	0.186	N/A^#^
δ IL-6	0.690	0.737	0.949	N/A^#^

*AqH* = aqueous humor; *GEE* = general estimating equation; *AL* = axial length; *VH* = vitreous humor; *IL-10* = interleukin 10; *IL-6* = interleukin 6; *δ* = VH concentration minus AqH concentration; *N/A* = not available

^*^ *P* < 0.05

^#^ The VH concentration was not included as an independent variable because it was involved in the calculation of the dependent variable (δ)

The influence of age, lens status and AL on the VH-AqH concentration difference was evaluated by GEE. δ IL-10 and δ IL-6 were not affected by age, lens status or AL, which indicated that the diffusion of IL-10 and IL-6 was less possibly affected by these factors but directly driven by the concentration gradient (Table 3).

### Correlations of clinical manifestations and cytokine levels

Vitreous haze was observed in all 74 eyes, of which the median grade was 2+ (range: 0.5+ to 4+). Intraretinal infiltration was observed in 33 (45%) eyes, while RPE involvement was observed in 52 (70%) eyes.

The correlations between IL levels and clinical manifestations were evaluated by stepwise linear regression. IL-10 in the AqH, IL-10 in the VH, IL-6 in the AqH and IL-6 in the VH were positively correlated with the presence of intraretinal infiltration (*P* = 0.038, 0.004, 0.020, and 0.003, respectively). Vitreous haze and RPE involvement had no significant correlations with IL levels (Table 4).

**Table 4:** Correlations of clinical manifestations on cytokine levels

Dependent variable	Significance of each clinical manifestation in regression		
	Vitreous haze	Intraretinal infiltration	RPE involvement
AqH IL-10	0.969	0.038*	0.732
VH IL-10	0.984	0.004*	0.659
AqH IL-6	0.576	0.020*	0.311
VH IL-6	0.984	0.003*	0.659

*RPE* = retinal pigment epithelium; *AqH* = aqueous humor; *VH* = vitreous humor; *IL-10* = interleukin 10; *IL-6* = interleukin 6

^*^ *P* < 0.05

Direct comparisons via the Wilcoxon rank-sum test indicated that the IL-10 levels of the eyes with intraretinal infiltrations were significantly higher than those without in both the AqH (median: 449.10 pg/mL vs. 141.08 pg/mL; *P* = 0.001) and VH (median: 2285.77 pg/mL vs. 825.48 pg/mL; *P* < 0.001), and so were the IL-6 levels in the AqH (median: 191.15 pg/mL vs. 47.61 pg/mL; *P* = 0.002) and VH (median: 231.15 pg/mL vs. 51.28 pg/mL; *P* < 0.001).

The positive rates of IL-related parameters were also compared between eyes with and without intraretinal infiltration (Table 5). The eyes without intraretinal infiltration had lower rates of AqH IL-10 > 30 pg/mL (32/41, 78%) and AqH ISOLD > 0 (31/41, 76%) when compared to those with (32/33, 97% and 32/33, 97%, respectively; *P* = 0.018 and 0.033, respectively). The positive rates of other parameters were not significant (Table 5).

**Table 5:** Positive ratio of parameters in eyes with and without intraretinal infiltration

Parameter	Positive ratio [positive eyes/total, (%)]		*P* value^#^
	Eyes with intraretinal infiltration	Eyes without intraretinal infiltration	
AqH IL-10 > 30 pg/mL	32/33 (97%)	32/41 (78%)	0.018*
VH IL-10 > 65 pg/mL	32/33 (97%)	35/41 (85%)	0.090
AqH IL-10/IL-6 > 1	26/33 (79%)	31/41 (76%)	0.747
VH IL-10/IL-6 > 1	31/33 (94%)	35/41 (85%)	0.238
AqH ISOLD > 0	31/33 (94%)	31/41 (76%)	0.033*
VH ISOLD > 0	32/33 (97%)	35/41 (85%)	0.090

*AqH* = aqueous humor; *VH* = vitreous humor; *IL-10* = interleukin 10; *IL-6* = interleukin 6; *ISOLD* = Interleukin Score for Intraocular Lymphoma Diagnosis

^#^ Pearson’s Chi-square tests

^*^ *P* < 0.05

### ***Influence of MYD88***^***L265P***^*** mutation on cytokines***

The differences in positive rates of all the IL-related parameters were of no significance between eyes with and without *MYD88*^L265P^ mutation (Table 6).

**Table 6:** Positive ratio of parameters in eyes with and without *MYD88*^L265P^ mutation

Parameter	Positive ratio [positive eyes/total, (%)]		*P* value^#^
	Eyes with *MYD88*^L265P^	Eyes without *MYD88*^L265P^	
AqH IL-10 > 30 pg/mL	46/54 (85%)	18/20 (90%)	0.719
VH IL-10 > 65 pg/mL	49/54 (91%)	18/20 (90%)	> 0.999
AqH IL-10/IL-6 > 1	42/54 (78%)	15/20 (75%)	> 0.999
VH IL-10/IL-6 > 1	48/54 (89%)	18/20 (90%)	> 0.999
AqH ISOLD > 0	46/54 (85%)	16/20 (80%)	0.724
VH ISOLD > 0	49/54 (91%)	18/20 (90%)	> 0.999

*AqH* = aqueous humor; *VH* = vitreous humor; *IL-10* = interleukin 10; *IL-6* = interleukin 6; *ISOLD* = Interleukin Score for Intraocular Lymphoma Diagnosis

^#^ Pearson’s Chi-square tests

## Discussion

In this study, we analyzed the correlations of IL-10 and IL-6 levels between the AqH and VH. Besides, we evaluated the correlation of clinical manifestations on the concentrations of ILs, and the positive rates of various IL thresholds for VRL screening.

Detecting ocular molecules systemically is challenging due to the blood-eye barrier's selective permeability [12]. Therefore, AqH is a minimally invasive approach to monitoring intraocular status and can thus facilitate both the diagnosis and management of various infections and inflammatory retinal diseases [13, 14]. For VRL, cytokine assay and detection of the *MYD88* gene mutation can be performed through intraocular fluids and are of great importance for preliminary diagnosis [11, 15–17]. Given the fact that droplet digital polymerase chain reaction (ddPCR) or sequencing has not yet been popularized or approved for clinical use in some regions, use of the cytokine assay is still a feasible and cost-effective choice for the screening of VRL [18]. However, whether the cytokines in the AqH can reflect retinal conditions in VRL is still unclear. In this study, we found that IL-10 levels and IL-10/IL-6 ratios were significantly lower in the AqH than in the VH, which was consistent with a higher diagnostic threshold of IL-10 in the VH [8]. On the other hand, the positive rate of IL-10/IL-6 > 1 in the AqH was lower than that in the VH, contrasting with the findings of Kuiper et al. in their previous study [19]. A possible reason was that IL-10 decreased more significantly from the vitreous to anterior chamber than IL-6 did. Therefore, IL-10/IL-6 in AqH might not be as reliable as other parameters with respect to sensitivity and should be used for a differential purpose. Additionally, our data indicated that the difference in cytokine concentrations between AqH and VH might not be affected by the condition of the lens or vitreous.

Besides, we further analyzed the correlations of clinical manifestations on IL-10 and IL-6 levels. Produced by malignant B cells through a variety of mechanisms, IL-10 is a key cytokine in the pathogenesis of B-cell lymphoma, and is considered a biomarker of tumor load [7, 20, 21]. Conventionally, vitreous cells are considered to be mainly composed of infiltrating malignant lymphocytes. However, our data showed that the degree of vitreous haze was not correlated with IL-10 level. A plausible reason is that reactive T cells are also an important part of the vitreous cells in eyes with VRL [22–24]; if the proportion of lymphoma cells is low, IL-10 may not correlate well with the extent of vitreous haze. Another finding was that, eyes without intraretinal infiltration had lower IL levels and positive rates of IL-10 > 30 pg/mL and ISOLD > 0 in AqH, which indicated that we should be cautious when ruling out the possibility of VRL via AqH cytokine assays for these patients. Close follow-up and repeated cytokine assays should be considered; ddPCR for *MYD88* c.794 T > C (p.L265P) mutation in AqH [15, 25], or a complete assay for immune mediators in AqH with machine learning based diagnostic algorithms could be attempted if necessary [26]; further IL measurement or cell-free DNA sequencing in VH could be helpful for these patients.

One limitation of this study was that we did not include uveitis samples and thus could not evaluate the specificity of IL measurements. It was because diagnostic vitrectomy was seldom performed for uveitis patients since many of them would have been diagnosed through other less-invasive approaches or diagnostic steroids therapy. Therefore, we focused on the AqH—VH correlation and cytokine—clinical manifestation correlation in confirmed VRL patients. Another limitation was that, due to the limited sample size and retrospective design, it was difficult to explore further predictors of abnormally low IL-10 levels in AqH among VRL patients; insufficient eyes with certain status (e.g., pseudophakic) might lead to an underestimation of these factors. We are in the midst of conducting a prospective cohort to include more uveitis cases and clinical factors in the hope of resolving these limitations in the future.

## Conclusions

IL-10 level and IL-10/IL-6 ratio was higher in the VH than in the paired AqH. The parameter, IL-10/IL-6 ratio in the AqH, was not as sensitive as the parameters in VH when used for VRL screening. In eyes without intraretinal infiltrations, AqH was less likely to be positive for IL-10 > 30 pg/mL and ISOLD > 0, thus leading to underdiagnosis. Close follow-up, repeated cytokine assays and VH sampling should be considered if VRL was to be ruled out.

## Data Availability

Not applicable. All the data for analyses are included in the article.

## Abbreviations

VRL: Vitreoretinal lymphoma
AqH: Aqueous humor
VH: Vitreous humor
IL-10: Interleukin 10
IL-6: Interleukin 6
ISOLD: Interleukin Score for Intraocular Lymphoma Diagnosis
OCT: Optical coherence tomography
RPE: Retinal pigment epithelium
AL: Axial length
95% CI: 95% confidence interval
GEE: General estimating equation
IOL: Intraocular lens
PCR: Polymerase chain reaction
